# Proteomic characterization of non-small cell lung cancer in a comprehensive translational thoracic oncology database

**DOI:** 10.1186/2043-9113-1-8

**Published:** 2011-02-28

**Authors:** Mosmi Surati, Matthew Robinson, Suvobroto Nandi, Leonardo Faoro, Carley Demchuk, Cleo E Rolle, Rajani Kanteti, Benjamin D Ferguson, Rifat Hasina, Tara C Gangadhar, April K Salama, Qudsia Arif, Colin Kirchner, Eneida Mendonca, Nicholas Campbell, Suwicha Limvorasak, Victoria Villaflor, Thomas A Hensing, Thomas Krausz, Everett E Vokes, Aliya N Husain, Mark K Ferguson, Theodore G Karrison, Ravi Salgia

**Affiliations:** 1 Pritzker School of Medicine, University of Chicago Pritzker School of Medicine, 924 E. 57th St., Chicago, IL 60637, USA; 2 Section of Hematology/Oncology, Department of Medicine, University of Chicago Pritzker School of Medicine, 5841 South Maryland Avenue Chicago, IL 60637, USA; 3 Department of Pathology, University of Chicago Pritzker School of Medicine, Chicago, IL, USA; 4 Department of Bioinformatics, University of Chicago Pritzker School of Medicine, Chicago, IL, USA; 5 Department of Pharmaceutical Sciences, University of Chicago Pritzker School of Medicine, Chicago, IL, USA; 6 Section of Hematology/Oncology, Department of Medicine, Northshore University Health Systems, 2650 Ridge Avenue, Evanston, IL, 60201, USA; 7 Section of Cardiac and Thoracic Surgery, Department of Surgery, University of Chicago Pritzker School of Medicine, Chicago, IL, USA; 8 Department of Health Studies, University of Chicago Pritzker School of Medicine, Chicago, IL, USA

## Abstract

**Background:**

In recent years, there has been tremendous growth and interest in translational research, particularly in cancer biology. This area of study clearly establishes the connection between laboratory experimentation and practical human application. Though it is common for laboratory and clinical data regarding patient specimens to be maintained separately, the storage of such heterogeneous data in one database offers many benefits as it may facilitate more rapid accession of data and provide researchers access to greater numbers of tissue samples.

**Description:**

The Thoracic Oncology Program Database Project was developed to serve as a repository for well-annotated cancer specimen, clinical, genomic, and proteomic data obtained from tumor tissue studies. The TOPDP is not merely a library--it is a dynamic tool that may be used for data mining and exploratory analysis. Using the example of non-small cell lung cancer cases within the database, this study will demonstrate how clinical data may be combined with proteomic analyses of patient tissue samples in determining the functional relevance of protein over and under expression in this disease.

Clinical data for 1323 patients with non-small cell lung cancer has been captured to date. Proteomic studies have been performed on tissue samples from 105 of these patients. These tissues have been analyzed for the expression of 33 different protein biomarkers using tissue microarrays. The expression of 15 potential biomarkers was found to be significantly higher in tumor versus matched normal tissue. Proteins belonging to the receptor tyrosine kinase family were particularly likely to be over expressed in tumor tissues. There was no difference in protein expression across various histologies or stages of non-small cell lung cancer. Though not differentially expressed between tumor and non-tumor tissues, the over expression of the glucocorticoid receptor (GR) was associated improved overall survival. However, this finding is preliminary and warrants further investigation.

**Conclusion:**

Though the database project is still under development, the application of such a database has the potential to enhance our understanding of cancer biology and will help researchers to identify targets to modify the course of thoracic malignancies.

## Background

There is considerable interest in understanding the pathophysiology contributing to cancer. One modern research paradigm suggests that understanding the genomic and proteomic alterations leading to cancer will lead to enhanced cancer prevention, detection, and targeted molecular therapeutic strategies. Capturing information regarding the nature of such alterations has been accelerated with the completion of the human genome project. Since then, scientists have been able to more rapidly and efficiently identify genetic alterations and consequently, the fields of genomics and proteomics have grown exponentially.

The identification of genetic and proteomic alterations, however, is only one part of the equation. It is essential to explore the functional relevance of these alterations as they relate to tumorigenesis in order to progress from an interesting observation to a beneficial therapeutic strategy. Growing interest in translational research has spurred the growth of biorepositories, such as the NCI OBBR [1], which are large libraries of banked biological specimens accessible to researchers for the study of a variety of diseases. Agencies from the national, state, private, and academic levels have all been actively engaged in the development of biorepositories to facilitate translational research.

A major limitation to conducting translational research is that basic science and clinical data are often stored in different databases [2]. This makes it challenging for basic science researchers to access clinical data to perform meaningful analysis. Additionally, research is often limited to readily available samples that may not be representative or sufficient in number to support or refute a specific hypothesis. The promise of modern biorepositories is that researchers can access large quantities of aggregated and verified data which can then be used to validate previously generated hypotheses or stimulate new hypothesis-driven studies [3].

The potential of modern translational research prompted the development of the Thoracic Oncology Program Database Project (TOPDP). The aims of this endeavor were to: (1) create a platform to house clinical, genomic, and proteomic data from patients with thoracic malignancies; (2) tailor the platform to meet the needs of clinical and basic science researchers; and (3) utilize the platform in support of meaningful statistical analysis to correlate laboratory and clinical information. The thoracic oncology database is unique from other biorepository systems because it is not merely a listing of available tissue samples but rather offers a glimpse into the proteomic and genomic characterization of these tissues.

Herein, we demonstrate how our thoracic oncology database can be used for data mining and exploratory analysis. This report will focus on the proteomic analysis of non-small cell lung cancer (NSCLC) identified within the database as a case study of how the database may be utilized. In 2010, there were estimated to be 222,520 new cases and 157,300 deaths from lung cancer [4]. Lung cancer has traditionally been dichotomized into two groups based on the histological features of the tumor: small cell and non-small cell lung cancer. NSCLC is the more common of the two sub-types of lung cancer, constituting 85% of cases [5,6]. Furthermore, studies have shown that NSCLC has less of a causal association with smoking than other forms of lung cancer [7] and therefore more than behavioral modification may be necessary to alter the course of this disease. Given the enormity of its impact, many in the research community are dedicated to better characterizing NSCLC.

Access to a comprehensive and validated database such as this is valuable to translational cancer researchers who may use this database to look at data from a large number of samples. Studies based on larger sample sizes may help validate hypotheses not generally supported based on experiments using limited samples. Furthermore, they may refute conclusions based on experiments which may have been biased and underpowered because of selected and limited samples. Analysis of aggregated data from databases such as ours will promote better understanding of complex diseases which in turn will lead to more clearly defined targets for cancer prevention, detection, and treatment.

## Construction and Content

### Subjects

#### Standard for subject enrollment

Clinical data were obtained from subjects enrolled under two IRB approved protocols: (a) Protocol 9571 - a prospective protocol designed to obtain tissue samples from patients who will have a biopsy or surgery at the University of Chicago Medical Center for known or potential malignancies, and (b) Protocol 13473 - a retrospective protocol to access tissue samples already obtained through routine patient care which have been stored at the University of Chicago Medical Center.

Under Protocol 9571, patients were consented during scheduled appointments in the thoracic oncology clinic. Patients who previously underwent biopsy or surgery at the University of Chicago were consented to protocol 13473 during subsequent clinic visits. Patients who were expired were exempt and their tissues were included under an exempt protocol.

#### Inclusion Criteria

Participants were selected if they were under the care of an oncologist at the University of Chicago Medical Center for a known or potential thoracic malignancy. Healthy controls were not included in this study. All subjects have or had a primary, recurrent, or second primary cancer that was pathologically confirmed. Subjects were adults over the age of 18 years.

#### Clinical Data Collection Protocol

Clinical information for consented or expired subjects was obtained through medical chart abstraction and entered into the database by the data curator. For quality assurance, clinical information was only added to the database following confirmation of the data in the patient's chart.

### Tissue Samples

#### Specimen Collection Protocol

Tissues of interest were malignant and originating in the thoracic cavity. Tissues containing a known or suspected malignancy were obtained during standard clinical care through a biopsy or surgery. No additional tissue, outside of what was necessary for a diagnostic workup, was specified under this protocol. The attending pathologist ensured that the amount of tissue collected was sufficient for clinical purposes. However, if additional tissue, not essential for the diagnostic process was available, this tissue was banked. When available, samples of both normal and tumor tissues were collected from each subject.

#### Pathology Tissue Banking Database

All records of biological specimens obtained under these protocols were maintained in the pathology department within eSphere, a pathology tissue banking database. The eSphere database was developed in order to catalogue detailed information about the biospecimens. The samples were described by procedure date, specimen type (fresh frozen, paraffin embedded), location of the tumor, type of tissue (tumor, non-tumor), and specimen weight. The eSphere database uses barcode identification in order to ensure patient confidentiality and to minimize errors. The system is password protected and it is only available to IRB approved users within the medical center.

#### Human Subject Protection

With the exception of expired patients for whom an IRB waiver was granted, only subjects for whom written informed consent was obtained were included in the study. The database is password protected and access was limited to clinical staff directly responsible for maintaining the database. Individual investigators performing molecular studies did not have access to patient identifying information (medical record number, name, date of birth). In compliance with HIPAA rules and regulations, all reports generated using the database were de-identified. The protocol was approved by the IRB at the University of Chicago.

### Development of the Database

#### Informatics Infrastructure

**Figure 1:**
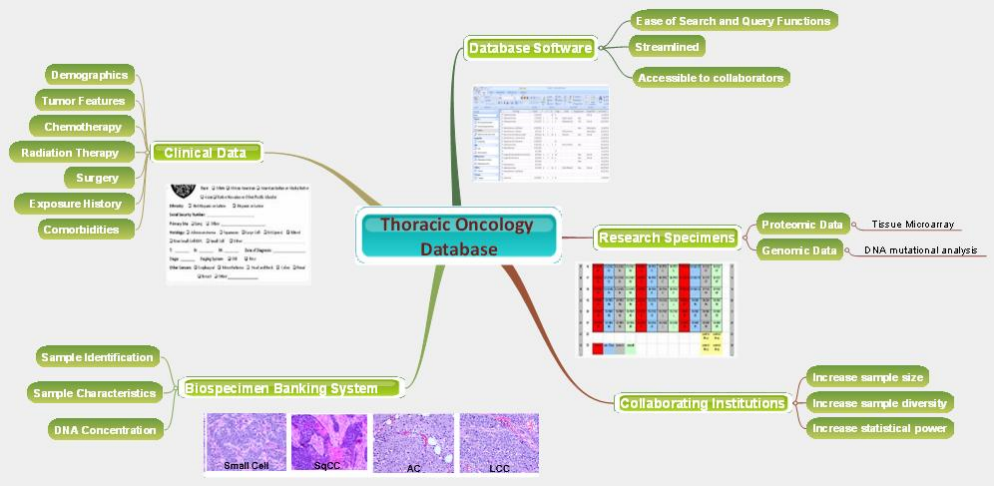
**Thoracic Oncology Program Database Project schematic**. Conceptual schematic depicting the multiple components contributing to the program.

To facilitate data storage and analysis, an informatics infrastructure was developed utilizing Microsoft Access as the primary repository of clinical and laboratory data (Figure 1). This program was selected based on a number of favorable characteristics including its ease of search and query functions. Other benefits of Microsoft Access include its large storage capacity and its ability to form relationships among multiple tables, thereby eliminating the need for data redundancy. Finally, Microsoft Access is readily available to most researchers. Though other database technologies are not necessarily prohibitive, it was important for the database team to select a program that could reduce barriers in collaborating with outside institutions who may also be interested in database initiatives.

#### Identification of Data Elements

The variables captured in the database were identified based on needs expressed by both clinical and basic science researchers. These elements respect the standards which emerged from the NCI Common Data Elements Committee [8]; however, they expand upon those standards to meet the needs of the research team. Variables of interest were established based on leadership provided by researchers from the department of hematology/oncology, pathology, surgery, radiation oncology, pharmacy, bioinformatics, and biostatistics. Standards used to establish the variables of interest were also based on precedent set by the Cancer Biomedical Informatics Grid (CaBIG) [9], the NAACCR [10] Data Standards for Cancer Registries, and the American Joint Committee on Cancer (AJCC) Staging Manual [11].

#### Development of Tables

Variables of interest were captured within four primary tables in the Access database: the Patients table, the DNA Specimens tables, the TMA table, and the Sample Data table. Each table captures different aspects of related information in a manner that reduces redundancy. For example, the main table in the database is the Patients table, which contains all clinically relevant information regarding the subject. This includes demographic information, clinically relevant tumor information including histology, stage, grade, treatment history, epidemiological factors, and patient outcome.

The DNA specimens table captures the genomic information characterizing mutations in tissue obtained from the subjects identified in the Patients table. This table is linked by the medical record number to the Patients table and thus there is no need to annotate tissue information such as histology, stage, and grade in the DNA Specimens table as that information is already captured.

**Figure 2:**
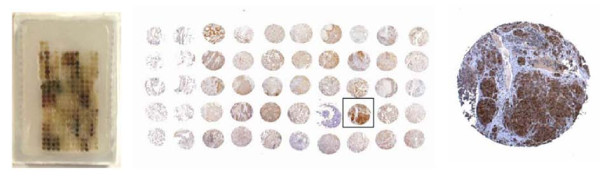
**Tissue Microarray (TMA)**. In a TMA, cores of tumor and adjacent normal tissue are removed from tissue embedded in paraffin blocks. Cores are arranged in an array and slices are stained using antibodies to assess the expression of proteins of interest.

The TMA table captures proteomic data from tissue samples that have been analyzed by tissue microarray (TMA). To facilitate the large-scale study of proteins expressed within the tumor, tissue microarrays were constructed as previously described [12]. The TMA were built using the ATA-27 Arrayer from Beecher Instruments. In brief, tissue cores (1-mm punch) from biopsied tumor and adjacent normal tissues were precisely organized into a grid and embedded in paraffin (representative image of TMA is shown in Figure 2). Paraffin blocks were separated so slices could be evaluated for the expression of various proteins using immunohistochemistry (IHC). IHC staining was performed using standard techniques and commercially available antibodies (see Appendix, Table 1).

**Table 1:** Source of Antibodies

Antibody	Vendor
c-Met	Zymed
p-Met 1003	Biosource
p-Met 1349	Biosource
p-Met 1365	Biosource
p-Met Triple	Biosource
HGF	R&D systems
Ronβ	Santa Crutz
p-Ronβ	Santa Crutz
Her3	Santa Crutz
EphA2	Santa Crutz
EphB4	Vasgen Therapeutics
Fibronectin	DAKO
β-catenin	Zymed
E-cadherin	Zymed
EzH2	Zymed
Snail	AVIVA Systems Biology
Vimentin	DAKO
Paxillin	Salgia Lab
GR	Novocastra
ERβ	Biogenex
PKCB-β1	Santa Crutz
PKCB-β2	GeneTex

Antibodies were purchased from the listed manufacturers.

IHC was scored on a semi-quantitative scale by a pathologist trained in this technique. All slides were reviewed by two independent pathologists. Each pathologist scored the tissue on a scale of 0 to 3 reflecting the degree of staining, with greater staining serving as a proxy for higher protein expression.

Two measures, the percent and intensity of IHC staining, were used to describe the level of protein expression in a tissue sample. Percent staining refers to the fraction of one core which stains positively for a particular protein. A core with less than 10% staining is scored a 1, between 11 and 50% staining is scored a 2, and greater than 50% staining is scored a 3. Intensity of staining compares the relative intensity of staining of one core of a TMA to that of a control core on the same slide. A score of 1 indicates faint staining, 2 indicates medium intensity staining, and 3 indicates dark staining. Furthermore, the pathologist is also able to visually assess the localization of predominant protein expression under the microscope and may categorize staining as being nuclear, cytoplasmic, or membranous. Thus, one protein may be characterized by multiple values.

Finally, the Sample Data table was developed in order to facilitate a link between the medical record number and the sample pathology number. The medical record number is unique to each patient while the sample pathology number is unique to each specimen. This table allows the researcher to rapidly determine the number of specimens catalogued in the database for each subject.

#### Query

With relationships established among the tables within the database, a query can be generated to combine related data. The query was performed by the data manager who exported data to the requesting researcher. It is important to note that exported information is de-identified by removing the medical record number, patient's name, and date of birth.

### Statistics

We have used the database to correlate proteomic information with clinical parameters for patients with non-small cell lung cancer. Within this database, a unique patient often had several TMA punches captured within the TMA table for a particular protein, reflecting the multiple types of tissue obtained for each patient. Therefore, samples were grouped according to tissue source: tumor tissue, normal tissue, and metastatic tissue for each patient with TMA data within the database.

An averaged protein expression score was calculated for all available normal and tumor samples for each patient (i.e., replicates of the same type of tissue for a given patient were averaged) for each protein studied in the TMA database. Averaged "tumor tissue" scores included all samples that were isolated from the center of the tumor. Averaged "normal samples" included samples described as "adjacent normal", "alveoli normal" and "bronchi normal".

A Wilcoxon signed-ranks test was used to compare protein expression between tumor and matched normal tissue for each patient. Differences were considered statistically significant for an α less than or equal to 0.05.

Heat maps were developed using R (R version 2.11.1, The R Foundation for Statistical Computing) to graphically display tumor protein expression so as to more readily identify variability in expression. Mean protein expression for a particular biomarker was calculated and was stratified by histology and also by stage. A heat map was generated for each parameter.

Proteins were clustered *a priori *in the heat maps by their functional families: receptor tyrosine kinase (RTK), epithelial mesenchymal transition (EMT), non-receptor tyrosine kinase (non-RTK), protein kinases (PK), and histone modifiers (HM) (Table 2). Groupings were not based on formal cluster analysis. Differences in protein expression among protein families were compared using Mann-Whitney U testing with significant differences occurring at a p-value ≤ 0.05.

**Table 2:** Protein Functional Families

RTK	EMT	NonRTK	PK	HM
Met	β-catenin	ER	PKC-β1	EzH2
Ron	E-cadherin	GR	PKC-β2	
EphA2	Fibronectin			
EphB4	Snail			
Her3	Vimentin			
HGF	Paxillin			

Proteins captured in the database were grouped by their functional families: Receptor Tyrosine Kinase (RTK), Epithelial Mesenchymal Transition (EMT), Non-receptor Tyrosine Kinase (NonRTK), Protein Kinase (PK), and Histone Modifier (HM).

Finally, tumor samples were independently studied to determine the impact of protein expression on survival. Multivariate survival analysis was performed using a Cox (1972) regression model in order to control for the influence of stage of diagnosis and age at diagnosis. Statistical analysis was performed using SPSS software (SPSS Standard version 17.0, SPSS).

## Utility

### Patient Characteristics

At the time of compilation of this study, a total of 2674 unique patients were entered into the database. Patients with non-small cell lung cancer comprise the majority of cases annotated within the database. Other cancers contained in the database include small cell lung cancer, mesothelioma, esophageal cancer, and thymic carcinoma, amongst others. Descriptive characteristics of the patients captured within the database were most often obtained retrospectively via chart abstraction. Demographic and clinical data for the 1323 NSCLC cases are summarized in Table 3.

**Table 3:** Patient Demographics

	Number of Cases (%)*		
	Entire Database	TMA only	Heat map only
**Gender**			
Male	688 (52)	63 (60)	46 (60)
Female	635 (48)	42 (40)	31 (40)
**Race**			
Caucasian	587 (44)	63 (60)	51 (66)
African American	377 (28)	34 (32)	23 (30)
Other	38 (3)	2 (2)	3 (4)
Non-Specified	321 (24)	6 (6)	n/a
**Histology**			
Adenocarcinoma	603 (46)	58 (55)	51 (66)
Large Cell Carcinoma	75 (6)	18 (17)	15 (19)
Squamous Cell Carcinoma	338 (26)	15 (14)	11 (14)
NSCLC Non-Specified	307 (23)	14 (13)	n/a
**Stage**			
I	379 (29)	49 (47)	37 (48)
II	123 (9)	12 (11)	8 (10)
III	261 (20)	32 (30)	27 (35)
IV	173 (13)	6 (6)	5 (6)
Non-Specified	384 (29)	6 (6)	n/a
**Vital Status**			
Alive	537 (41)	32 (30)	24 (31)
Deceased	452 (34)	71 (68)	53 (69)
Unknown	334 (25)	2 (2)	n/a
**Mean Age at Diagnosis**	64 years	61 years	61 years
**Median Survival**	17 months	16 months	17 months
**Total NSCLC Cases**	**1323**	**105**	**77**

*Due to rounding, percentages may not sum to 100.

To date, 1323 NSCLC patients have been captured in the database. A subset of these have TMA data (n = 105) and a further subset of patients were included in the heat map analysis.

### TMA and Analysis

A total of 867 cores from 105 unique patients were analyzed for their level of expression for 17 different proteins using tissue microarray (TMA). Demographic and clinical data for the NSCLC patients with proteomic data is summarized in Table 3. These patients are comparable to the NSCLC dataset in terms of gender, racial, histologic, and stage composition, vital status, mean age at diagnosis, and median survival.

For any given protein biomarker, the database contained tumor and corresponding normal data for 50 to 100 patients. Though only 17 proteins were included in this analysis, a total of 33 protein biomarkers were evaluated. This is due to the fact that for certain proteins, different protein localizations (nuclear, membranous, and cytoplasmic) were compared between tumor and matched normal samples. Furthermore, for a given protein, both a protein percent staining score and a protein intensity staining score may have been calculated. All of these values serve as a proxy for the degree of protein expression and thus are included in the analysis.

The protein expression of tumor samples was compared with protein expression from normal tissue from the same patient. There were 15 potential biomarkers for which expression was significantly higher in tumor tissue (p < 0.05), 2 protein biomarkers for which expression was greater in normal tissue, and 16 protein biomarkers for which expression was not significantly different between the two tissue types (Table 4).

**Table 4:** Comparison of Protein Expression between Tumor and Normal Tissue

Tumor > Normal	Normal > Tumor	Tumor = Normal
**c-Met Cytoplasmic**	**c-Met Membranous**	**p-Met 1003 Nuclear**
**p-Met 1003 Cytoplasmic**	**c-Met Nuclear**	**p-Met 1365 Nuclear**
**p-Met 1349 Cytoplasmic**		**p-Met Triple Nuclear**
**p-Met 1349 Nuclear**		**Ron Membranous**
**HGF Cytoplasmic**		*Fibronectin Intensity*
**p-Ron Cytoplasmic**		*Β-catenin Intensity*
**p-Ron Nuclear**		*E-cadherin Intensity*
**Her3 Cytoplasmic**		*Snail Percentage*
**Her3 Nuclear**		*Snail Intensity*
**EphA2**		*Vimentin Percentage*
**EphB4**		*Paxillin*
*Fibronectin Percentage*		GR
*β-catenin Percentage*		ER β
*E-cadherin Percentage*		*PKC-β1*
EzH2 Percentage		*PKC-β2*
		EzH2 Intensity

Protein expression was compared between tumors and matched control tissue. Certain proteins were found to differentially expressed, while others were not. These differences were statistically significant. Proteins are organized by functional families represented by different fonts.

A few interesting trends emerged. For c-Met, there was greater expression of the protein in the tumor than in the matched normal tissue for the cytoplasmic localization of the protein but the reverse was true for the membranous and nuclear distributions. For p-Met 1003, the cytoplasmic distribution was greater in tumor than in matched normal tissue, but there was no difference in p-Met 1003 nuclear expression. Finally, for p-Met 1349, p-Ron, and Her3, tumor expression was greater for both the cytoplasmic and nuclear localizations than matched normal tissue. This suggests that though protein expression may be generally greater in tumor tissue, it may selectively be observed in different parts of the cell.

For protein biomarkers such as fibronectin, ß-catenin, E-cadherin, and EzH2 the relative percentage of the tumor core which stained positively for a given biomarker was greater than matched normal tissue. However the intensity of biomarker staining did not differ. There is evidence to suggest that percentage staining may be a marker which is better correlated with relevant tumor endpoints and thus may be preferred to intensity values [13]. Differential percent staining but the lack of a differential intensity staining suggests that tumor tissue is globally producing more of a given protein rather than in focal areas of tumor.

### Heat map analysis

Data from a total of 77 patients with tumor protein expression data, histologic categorization, and stage categorization were included in the heat map displays. These patients were a subset of the 105 patients included in the TMA analysis and were selected because they had protein expression data within each of the protein families. These patients are comparable to the TMA analysis group in terms of gender, racial, histologic, and stage characterization, vital status, mean age at diagnosis, and median survival (Table 3).

**Figure 3:**
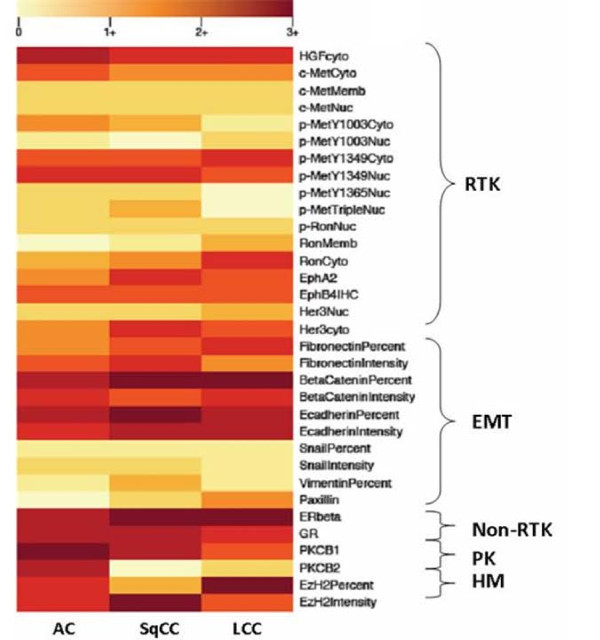
**Heat map based on tumor histology**. Averaged tumor protein expression values for given proteins are stratified by tumor histology: adenocarcinoma (AC), squamous cell carcinoma (SqCC), and large cell carcinoma (LCC).

**Figure 4:**
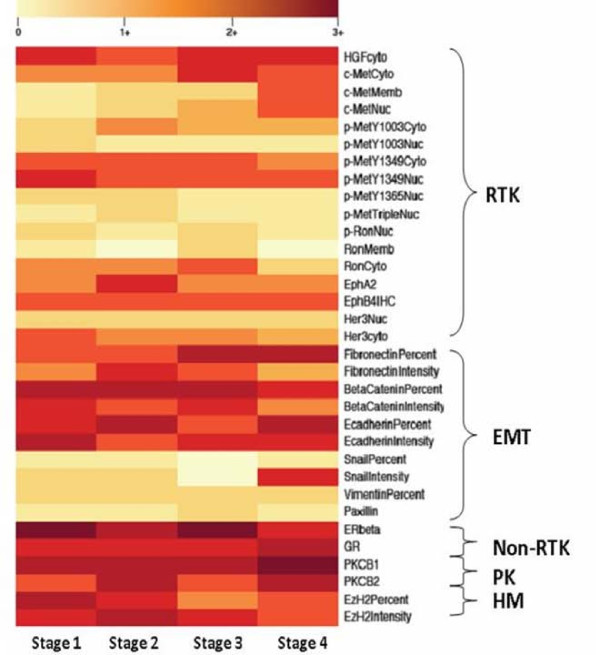
**Heat map based on tumor stage**. Averaged tumor protein expression values for selected proteins are stratified by tumor stage at diagnosis.

Based on the heat maps, differential expression patterns were noted. Firstly, when protein expression was categorized by histology, the non-RTK, PK, and HM families of proteins tended to be more highly expressed than RTK and EMT proteins in tumor tissue (p = 0.05) (Figure 3). When the proteins were separated by stage, a similar pattern emerged (p = 0.00) (Figure 4). Notably, these same patterns were reproduced when analyzing matched normal tissue (p = 0.001 and p = 0.002, respectively). This may be due to a few reasons. Differences in antibodies used to stain for various proteins may provide a technical consideration when comparing expression between different proteins. Furthermore, as there were more members of the RTK and EMT families than the other groups, averaged RTK and EMT could have lower values due to data reduction.

In addition, there was a trend towards higher protein expression in adenocarcinoma and large cell carcinoma than in squamous cell carcinoma; however, this difference was not statistically significant (one way ANOVA; p = 0.16). This was suggestive of but not diagnostic for global protein over-expression within these histologies. There was no difference among the stages related to overall protein expression (one way ANOVA; p = 0.92).

### Survival Analysis

To study the relationship between protein expression and survival in non-small cell lung cancer, expression data from 33 protein biomarkers were studied using both univariate and multivariate analyses. Of the proteins studied, only one was found to have a nominally statistically significant association with survival, the glucocorticoid receptor (GR).

**Figure 5:**
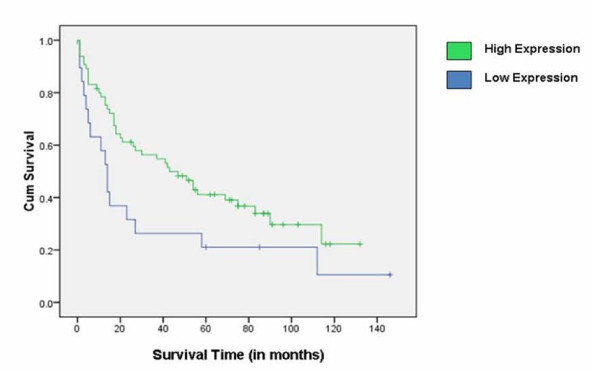
**Kaplan Meier Survival Curve for GR**. Survival curves were dichotomized on the median expression value of the Glucocorticoid receptor (GR). Higher expression of GR was associated with greater overall survival. Tick marks represent censored data points.

In univariate survival analysis, a cumulative survival curve was calculated using the Kaplan-Meier method. Protein expression was stratified into two categories: under- and over-expression. Protein expression was dichotomized at the median tumor GR expression value of 2.13. The survival difference between the two protein expression curves was assessed using a log-rank test. The median overall survival time for patients with GR under-expression was 14 months, while the median overall survival time for patients with GR over-expression was 43 months. The difference in survival time between the two groups was statistically significant (p = 0.04) (Figure 5).

Since known prognosticators could confound the association between protein expression and survival time, a multivariate Cox regression model was used to predict the impact of protein expression on survival after controlling for stage of disease and the patient's age at diagnosis.

There were 93 patients for whom the expression of the protein GR had been studied. Using a Cox regression model, a statistically significant hazard ratio of 0.76 (95% CI: 0.59, 0.97) was calculated (p = 0.03). Therefore, GR over-expression was associated with increased patient survival. Similar findings were previously noted in patients with advanced non-small cell lung cancer [14]. It should be noted, however, that after adjusting for multiple comparisons (33 protein biomarkers were evaluated), this finding does not reach statistical significance. Thus these results should be viewed as hypothesis-generating only, in need of further confirmation in an independent dataset.

## Discussion

Given that lung cancer is the leading cause of cancer related death in the United States, there is tremendous interest in identifying markers which may not only help to better elucidate oncogenic pathways but also lead to clinically relevant targets involved in the diagnosis and treatment of this disease. Though much research has been invested into the discovery of such biomarkers, often they have proved to be of limited clinical utility [15].

While genomics research continues to play an important role, increasing emphasis has been placed on proteomics in the area of biomarker research [15]. Often proteomic studies will focus on the expression of one protein of interest or one family of proteins and will relate these outcomes to relevant clinical endpoints [14,16-19]. While this is important work, it is our belief that by developing a database in which multiple biomarkers and their interactions may be studied simultaneously, we will be better equipped to understand the complex interplay among various proteins and its relation to oncogenesis. This may lead to the hypothesis generation necessary to identify a relevant target or multiple targets in the cancer pathway.

A view of the descriptive data presented in the heat maps suggests that proteins in the non-RTK, PK, and HM families are more highly expressed in tumor tissues than proteins from the RTK and EMT families. However, when the comparison is made between tumor and normal tissues, predominantly RTK proteins appear to be differentially expressed between the two tissue types. This suggests that though non-RTK, PK, and HM proteins may be more highly expressed globally, RTK proteins may make for better clinical targets because of their discrepant expression. This finding further validates the notion of MET [20] as a therapeutic target in lung cancer and should reinforce research regarding this potential biomarker in the treatment of non-small cell lung cancer.

The data analyzed here highlights the potential of the TOPDP as a translational research tool. The data demonstrates that large amounts of information can be readily accessed and analyzed to support translational efforts. The formation of such a system promotes both hypothesis-driven and exploratory studies. However, it is important to understand the limitations of this database project in its present form. Furthermore, additional studies will be necessary to determine the functional importance of identified proteins.

A major consideration to make when interpreting the results of the exploratory analyses done on the tissue microarrays has to do with sample size. While the database has information on over 2500 patients, it is still relatively small compared with most databases. Furthermore, since each protein biomarker studied may have only had expression data from 50-100 patients for a particular type of cancer, there may not be a large enough sample size to detect the impact of protein under- or over-expression on clinical endpoints. Another limitation is that tumor tissues were not studied for every protein of interest. Any given tumor sample may have only been studied for the expression of a limited number of proteins. Though cumbersome and costly, it would be valuable to have proteomic analysis for every protein of interest for every patient within the database.

Given its focus on malignancy, an inherent caveat of the database is the lack of true normal controls. It can be argued that tissue adjacent to tumor tissue may be subject to stresses different from other tissues and thus does not represent true normal tissues. While this may be true, it is less common to have biopsy or surgically resected tissue from an individual outside the course of their cancer workup and treatment. Although it may be beneficial to bank normal tissue from healthy individuals, this is not a reasonable endeavor at this time. The caveat of "normalcy" is important and warrants consideration in the process of comparing "tumor" and "normal" tissues within our biorepository. It is also important to note that since tissues were obtained during the course of a patient's diagnostic or therapeutic care, not all patients had both "tumor" and "normal" tissue samples available in the biorepository.

As this has been both a retrospective and prospective initiative, the shortcomings of chart abstraction have become evident. The availability of dictated clinic notes is variable as many paper notes have not yet been entered into the electronic medical record system. This limits the amount of data that can be entered in the database by the data curator. In addition, if the physician dictating clinic notes did not describe epidemiological factors such as smoking history, these variables were not documented for all patients. Fortunately, moving forward, detailed questions will be asked of patients enrolled in the prospective protocol and as such, more detailed information will be available.

Another limitation of the database is that detailed vital status information is not available on all patients. Since patient medical charts are not linked to external sources, if the patient expires outside of our institution, our system is not aware of this event. Thus some patients may incorrectly be listed as living. In order to obtain more accurate vital status information, our team has used the Social Security Death Index [21] to periodically determine the vital status of patients within our database. Though efforts are made to update the database every six months, it is important to have an automated means of updating vital status. Similarly, for the purposes of survival analyses, the date of last contact with our institution was used to censor living patients. Given that a patient may have transferred care to an outside institution and have died, censoring the survival time at the date of last contact may bias our estimates.

Finally, while the database reasonably captures information about a patient's treatment course, it could do so with greater detail. Differences in the types and timing of therapy may serve as important covariates in multivariate analyses. It is important to capture relevant detail regarding the complexity of a patient's treatment course. The database team is already in the process of advancing the database to make this capability possible.

## Conclusion

The database developed as part of the Thoracic Oncology Program Database Project serves as an example of the collective effort towards advancing translational research. This database is unique in that it is not merely a list of stored specimens but rather proteomic and genomic characterizations are captured within the database as well. In this manner, proteomic data can be analyzed in aggregate and is not limited to the small sample sizes common to most basic science research. With additional sample size, data is more robust and real trends may be identified.

In an effort to further increase sample size, the standard operating procedure and database template has been made available online at http://www.ibridgenetwork.org/uctech/salgia-thoracic-oncology-access-template. By freely sharing the design of this database with collaborators at outside institutions, it is anticipated that they may develop their own database programs. The development of such databases requires the establishment of clearly defined protocols detailing methods by which tissue samples are collected and clinical information are annotated. This will in turn ensure high specimen quality as well as consistency of clinical information obtained. With variables captured identically across geographic locales, data may be reliably combined [22]. There are many benefits for inter-institutional collaboration. Not only will this increase sample size and increase statistical power for proteomic and genomic studies [23], this will also increase the diversity of the patient sample captured within the database. In this manner, disparities in cancer outcomes may be further explored.

Though promoting collaboration is an important priority of the database team, the decision was made not to make this a web-based database. Freely allowing outside collaborators to contribute to one shared database raises important IRB and intellectual property related concerns. Thus, this database is maintained within our institution and when outside collaborators have developed their own databases and would like to share data, appropriate steps can be taken with specific institutional regulatory bodies.

Through the established infrastructure of the Thoracic Oncology Program Database Project, clinical and basic science researchers are able to more efficiently identify genetic and proteomic alterations that contribute to malignancy. The evolution of bioinformatics in practice will further promote the development and translation of important laboratory findings to clinical applications. Accurate, accessible, and comprehensive data facilitates better research and will promote the development of more effective solutions to complex medical diseases.

## Abbreviations

AJCC: American Joint Committee on Cancer; CaBIG: Cancer Biomedical Informatics Grid; EMT: Epithelial Mesenchymal Transition; HIPAA: Health Insurance Portability and Accountability Act; HM: Histone Modifier; IHC: Immunohistochemistry; IRB: Institutional Review Board; NAACCR: North American Association of Central Cancer Registries; NCI: National Cancer Institute; Non-RTK: Non-Receptor Tyrosine Kinase; NSCLC: Non-Small Cell Lung Cancer; OBBR: Office of Biorepositories and Biospecimen Research; PK: Protein Kinase; RTK: Receptor Tyrosine Kinase; TMA: Tissue Microarray; TOPDP: Thoracic Oncology Program Database Project

## Competing interests

The authors declare that they have no competing interests.

## Authors' contributions

MS, MR, SN, CD, and CER drafted the manuscript. MS, MR, SN, LF, CD, CER, NC, and SL are involved in the design and the maintenance of the database. MS, MR, SN, CD, CER, SL, MC, CK, EM, and TGK are part of the advisory committee involved with database development, transition, and outside collaboration. SN, CER, RK, BDF, RH, TCG, and AKS participated in data generation. TK and AH participated in TMA analysis and support from the department of pathology. TCG, AKS, NC, VV, TAH, EEV, MF, and RS provided clinical support. TGK assisted with the interpretation of the results and manuscript preparation. RS has been integral to the conceptualization and development of the database project, as well as overall manuscript preparation. All authors read and approved the final manuscript.
